# Anxiety and Worry about Six Categories of Climate Change Impacts

**DOI:** 10.3390/ijerph21010023

**Published:** 2023-12-23

**Authors:** Alan E. Stewart, Harrison E. Chapman, Jackson B. L. Davis

**Affiliations:** College of Education, University of Georgia, Athens, GA 30602, USA; harrison.chapman25@uga.edu (H.E.C.); jackson.davis26@uga.edu (J.B.L.D.)

**Keywords:** climate change, anxiety, worry, climate change impacts, consequences of climate change, psychological distance, sustainable behaviors

## Abstract

The occurrence of severe and extreme weather events that have been attributed to a changed climate system and the widespread dissemination of the impacts of these events in the media can lead people to experience concern, worry, and anxiety, which we examined in two studies. In Study 1, we observed that people more frequently expressed worry than anxiety about the impacts of climate change in six areas. People were more frequently worried and anxious about the effects of climate change on future generations and about societal responses (or lack of a response) to climate change. The levels of anxiety that people expressed were significantly higher than the worry people reported when anxiety was their modal response. In Study 2, we observed that both climate change worry and anxiety were negatively correlated with psychological distance from climate change. Overall, climate change worry and psychological distance significantly predicted climate-sustainable behaviors. Our study was among the first to use developed measures of climate change worry, anxiety, and psychological distance to examine peoples’ responses across some of the possible impact and consequence areas of climate change.

## 1. Introduction

The sixth and most recent Assessment Report of Impacts, Adaptation, and Vulnerability of the Intergovernmental Panel on Climate Change IPCC, [1] revealed high to very high risks from each of the IPCC five Reasons for Concern (RFC): (1) unique and threatened systems (e.g., biodiversity, arctic, and reef systems); (2) extreme weather events (e.g., heatwaves or extreme rainfall); (3) distribution of impacts (e.g., agriculture, water stress); (4) global aggregate impacts (economic and biodiversity damages); (5) large-scale singular events (e.g., Arctic, Antarctic, and Greenland ice systems). This represents a significant increase in climate change risks over the Fifth Assessment Report in 2014 [2]. In addition, the IPCC generally expects negative impacts in both the near term of 2021–2040 and the extended term of 2041–2100 on (1) water availability and food production; (2) physical health and mental well-being, and (3) cities, settlements, and infrastructure (e.g., inland and coastal flooding that damages infrastructure and economic sectors) [1]. Although water availability may fluctuate and be more abundant in some areas under climate change, the impacts beyond this tend unequivocally to be both negative and global. Regions where historical, political, and economic vulnerabilities overlap with increasing climate change risks, such as small island nations and some equatorial regions, are likely to be hard-hit by climate change events.

The IPCC Assessment Report, along with ongoing occurrences of climate and weather events, has continued to raise peoples’ awareness of climate change through the direct experience of their effects and/or through learning vicariously about climate change impacts through the media [1,3,4,5]. Some climate change impacts may be more present, close, direct, immediate, and concrete, while other impacts may be distant with respect to regions and the timescales involved, somewhat diffuse, and lend themselves less to direct experience.

Within this article, the authors present the results of two studies that investigated the nature of peoples’ psychological responses (from lack of any concern to anxiety) to six general categories of possible climate change impacts. The first study assessed peoples’ modal psychological responses to each of the six categories of climate change impacts. The second study examined the relationships of scores on measures of climate change worry, climate anxiety, and psychological distance from climate change with responses to each of the six possible impact areas of climate change. In the sections that follow, the authors discuss first the ongoing costs and losses associated with weather and climate disasters and how media reports about such events (and the IPCC reports) can focus attention on climate change. Second, the authors discuss *concern* as a possible response to climate change. This is followed in a third section by a review of recent research on climate change *worry*. The fourth section discusses research on climate *anxiety*. These responses (concern, worry, and anxiety) are related but distinct psychological responses, as will be discussed in Section 1.4, Section 1.5 and Section 1.6. After this literature review, the methods and results of Study 1 are presented.

### 1.1. Categories of Climate Change Impacts and Consequences

The most recent reports of weather- and climate-related disasters are generally consistent with the unfolding outcomes delineated in the IPCC assessment [1]. The year 2022 was the seventh most costly year on record for natural disasters in the United States [3,4]. The ten most costly disasters in 2022 produced USD 313 billion in losses and resulted in the loss of over 2000 lives [3]. Over time, both the number of billion-dollar loss events and the amounts of losses due to weather and climate disasters have increased [3,4]. Most recently, since January 2023, the United States has experienced a record 23 events that each have resulted in over USD 1 billion in losses [5].

Over time, researchers have identified several domains or areas in which global climate change may have impacts or consequences, which are related to some of the RFCs from the IPPC. One set of wider concerns people express about climate change involves what Abate [6] refers to as the *voiceless*—the natural environment, marginalized people or cultures, and future generations of people. People who possess pro-environmental attitudes express concerns about the *voiceless* within the context of climate change [6]. This relates to the first (i.e., threatened ecosystems), third (distribution of impacts), and fourth (global aggregate impact) RFCs from the IPCC [1]. Another area of concern involves the ability to live and work in the future under the regime of a changed climate, which relates to first, second (i.e., extreme weather events), and third RFCs [1,7,8]. As extreme events affect areas, resources, and economic activities, impacts on lives and livelihoods form a third category of concern and relate to the second, fourth, and fifth (i.e., large-scale singular events) RFCs [1,8]. Another wide-ranging concern involves large-scale disasters that bring massive destruction and irrevocable changes and losses in the physical and ecosystemic, social, and economic domains [7,8,9,10]. Concerns about the occurrence of such apocalyptic events appear to go beyond individual weather events to encompass multiple system failures as climate-related consequences spread and cascade [10,11]. These impacts and consequences relate to the second, third, fourth, and fifth Reasons for Concern from the IPCC [1]. A final area of concern involves the societal responses (governments, organizations, institutions, citizenry) in an effort to mitigate the causes of climate change and adapt to its effects [12,13]. The IPCC report does not address this impact area specifically, but conceptually, this concern relates to responses to global impacts (RFC #4) [1]. Consistent with this literature and the IPCC report, Soutar and Wand [14] conducted a scoping review of qualitative research on the variables that can influence the development of climate-related worry and anxiety. Soutar and Wand identified and examined several of the same major impact and consequence areas, such as the occurrence of large-scale disasters, effects upon peoples’ livelihoods, and concern for future generations of people [14].

### 1.2. Weather and Climate Impacts in the Media

Because extreme weather events, disasters, and the releases of important documents such as the most recent IPCC report tend to be focusing events, it is not surprising to see corresponding fluctuations and increases in the media’s coverage of climate change [15,16,17]. Such coverage is significant because it raises awareness about climate change and promotes discourse about the issue [18,19]. Societal dimensions of climate change tend to receive the most coverage, especially in the global South, with correspondingly less coverage on climate science, ecological or environmental aspects, or effects on future generations [16,20]. Coverage of severe weather outbreaks that are putatively attributed to climate change can have emotional impacts on those who consume the coverage [21]. Concern, worry, and anxiety may all increase as people consume media coverage of climate change and then seek more information as they attempt to cope with negative feelings [22,23]. Beyond this, media coverage of climate change increasingly reflects the politicization and polarization of contemporary issues [24]. Chinn, Hart, and Soroka examined the polarization in the media as different political factions have attempted to use the media to advance their narratives, especially as this relates to casting doubt about the sources and nature of climate change and misrepresenting climate science [24]. To some extent, media coverage of extreme weather events may also report on climate change in an attempt either to understand or to possibly attribute the extreme events to changes in the climate system [25,26].

### 1.3. Psychological Distance and Climate Change

The occurrence of weather and climate events, along with reporting about them in the media, are related to the concept of psychological distance [27]. Psychological distance is the perceived distance or separation between an individual’s current self and situation and the self’s relationship to different dimensions, including temporal, spatial, social, and hypothetical distance. According to Trope and Liberman, “Psychological distance refers to the perception of *when* an event occurs, *where* it occurs, to *whom* it occurs, and *whether* it occurs” [27], p. 442. Psychological distance is related to Construal Level Theory, which pertains to how a person thinks about or represents the event that may have relevance for them. “Construal levels refer to the perception of what will occur: the processes that give rise to the representation of the event itself” [27], p. 442. Events that are psychologically close are experienced in concrete and practical terms. Those events that are psychologically distant are engaged in progressively more abstract levels.

In the context of climate change, psychological distance is a multidimensional, context-specific, and dynamic construct that may yield varying results when researched [28,29]. Generally, it has been observed that when climate change impacts have been perceived as proximal and more immediate (i.e., small psychological distance), people are more willing to perform pro-environmental behaviors [29,30,31,32]. Perhaps because of the context-specific nature of psychological distance, some researchers have observed that psychological distance may not reliably relate to peoples’ perceptions about the impacts of climate change [32,33,34]. The relationship of psychological distance to climate change impact perceptions also may be affected by individual difference variables such as trait empathy [35], political orientation [36], and views about the roles of science [37].

Regarding emotions, reduced psychological distance to climate change impacts have been associated with increases in concrete emotional experiences such as anger, fear, sadness, and guilt. [35]. Stewart observed that people were more psychologically attentive and mindful of the weather if they experienced a weather event that produced property damages or injuries [38]. Similarly, Li observed that local and daily temperature variations influenced peoples’ concerns about global warming [39]. Thus, the direct experience of weather or climate events and/or exposure to media coverage of those events may make these events psychologically close and elicit a range of psychological experiences in people, ranging from a complete lack of awareness to concern, worry, or anxiety. In this regard, we consider psychological distance an antecedent of worry and anxiety responses. We discuss these possible responses below.

### 1.4. Concern about Climate Change

Researchers in both climate science and in the societal impacts of climate change have used the term *concern* to convey the degree of interest, care, importance, or curiosity that exists about the processes that underlie climate change and its consequences [40,41,42,43]. The IPCC has employed the Reasons for Concern framework (reviewed above) since the Third Assessment Report in 2001 to describe the physical basis of climate change [44]. Similarly, reports about the public perception of various aspects of climate change use *concern* and rating scales of degrees of *concern* to gauge respondents’ attitudes, feelings, and behaviors [41]. The Pew Research Center 2015 survey is a noteworthy example of this approach [45].

Within the context of the psychology of climate change and for the purposes of this article, the authors define concern about climate change as the extent to which a person thinks that climate change is important and that it is something that they care about. Climate change concern described in this way is like more general environmental concern that involves “the degree to which people are aware of environmental problems and support efforts to solve them and/or indicate a willingness to contribute personally to their solution” [46], p. 485. This definition of *concern* is also consistent with ordinary language usage and with the conceptions of it that have appeared in the literature [47,48]. Importantly, van der Linden has developed a hierarchy of concern for climate change that involves elements of both the likelihood of climate change impacts occurring and the perceived seriousness of the impacts [48]. A person who is concerned about climate change impacts may express this concern when asked about it or when a situation arises (e.g., a media report) that makes them aware of a recent climate development, for example. Concern is a necessary but insufficient element of worry about climate change [47,48] because it lacks the motivational and emotional elements that are features of worry. [49,50]. A concern may persist over time, but as a concern, it does not involve negative, troubling feelings. Beyond this, a high and ongoing degree of concern about climate change or its aspects may transition into persistent and emotionally troubling thoughts that essentially become a *worry* for the person [48].

### 1.5. Worry about Climate Change

Beyond peoples’ concerns, psychologists have begun to study the range of possible mental health effects associated with climate change [15,51,52,53,54,55,56,57,58,59,60,61]. Trauma, grief, bereavement, and loss have been associated with the experience of severe or extreme weather events [61,62,63,64,65]. Depression and anxiety also may be common in the aftermath of climate-related disasters [61,66,67]. Further, the anticipation of future or subsequent events and concerns about both proximal and wider climate change impacts, as reviewed above, can give rise to worry, anxiety, and related emotions of eco-anxiety, eco-guilt, and eco-grief, among other responses [51,57,58,61,68]. Worry and anxiety are a focus of this article, in part because they have received the most attention in the mental health literature on climate change. Relatedly, worry and anxiety represent among the most common psychological responses to both past and possible future climate-related events [15,69].

For the purposes of this article, and consistent with previous research [68], climate change worry means that a person has concerns about climate change that are troubling to them and that give rise to negative thoughts or feelings; worry is realistically based, and is usually temporary. Worry by itself, especially about climate change, is normal and understandable [70,71], and can motivate people to act about climate change [72]. Concern and worry are related in that they both pertain to cognitions or beliefs about something, which in the present context involve the climate and climate change. For worry, the added components of troubling thoughts and feelings can be more challenging for the person to manage compared to a concern [68,72].

The concept of climate-related worry is anchored in the affective and clinical literature that has examined worry [68]. In general, worry involves negative verbal-linguistically-based thoughts and minimal imagery [73,74]. Worry can indicate engagement in mental problem-solving for situations that have uncertain or possibly negative consequences [75]. Worry also encompasses apprehension about the occurrence of negative events in the future [76,77]. People who worry have difficulties remaining calm and may exhibit signs of stress, such as tension, nervousness, or irritability [68,78,79,80]. Worry may become problematic if it becomes generalized, repetitive, and outside of the person’s ability to control [76,78,79,80,81,82,83].

The first author has developed a measure of climate change worry (the Climate Change Worry Scale, CCWS) [68] that consists of ten items encompassing different domains of mostly proximal effects of climate change. Scores on CCWS are correlated with political orientation, fear of weather, fears about storms, stress, depression, and anxiety measures. Other researchers who have investigated climate change worry with the CCWS have reported that the measure exhibited statistically significant correlations with climate change anxiety, stress, pro-environmental behaviors, the New Social Paradigm subscale, and the Dominant Social Paradigm subscale of the New Ecological Paradigm, climate change information, and political engagement [84]. Researchers in Israel examined how climate change exposure, climate risk perceptions, and self-efficacy, among other variables, contributed to climate change worry [85].

### 1.6. Climate Change Anxiety

Climate change worry that becomes persistent in a person’s life and additionally involves increases in their autonomic arousal (increases in heart rate, breathing, sweating, and tension) can lead to climate anxiety [68]. Climate change worry is thus one of the components, along with arousal, that make up climate change anxiety. More specifically, climate change anxiety involves “anxiety which is significantly related to anthropogenic climate change” [86], p. 3. Clayton provided a more comprehensive definition of climate anxiety as “anxiety associated with perceptions about climate change, even among people who have not personally experienced any direct impacts” [57], p. 2. Climate anxiety represents a specific form of the broader construct of eco-anxiety, which itself is a general anxiety about harm or preventable changes that occur to the environment [56]. Climate anxiety has become a major focus of interest and investigation in the research and mental health communities [87,88,89,90,91,92,93,94,95,96]. Increasingly, people are attributing their climate change-related distress to climate anxiety [97].

Clayton and Karaszia observed that climate anxiety was related to emotional responses to climate change and developed a climate change anxiety measure that captures both emotional impairment and disruptions in relationships and life activities (Climate Change Anxiety Scale, CCAS) [58]. Beyond this work, the CAS has generated much interest in research. For example, several studies have sought to validate the CAS in different cultural samples and languages. Feather and Williams [88] validated the scale in Australia and New Zealand, noting a reasonable global fit of the items while suggesting some areas for improving the scale. International non-English interest in the CCAS is significant, as Wullenkord et al. [96] translated and validated the CAS through a German population quota sample, Innocenti et al. [91] translated and validated the CAS through an Italian population sample, and Daouda et al. [92] translated and validated the scale through a French population sample. Researchers have applied the CCAS to both European and African French-speaking participants [90], noting no significant difference in climate anxiety frequency per the scale between the two groups of participants. The CCAS has also been used with Filipino participants, investigating the link between climate anxiety and larger mental health [93] and defining the potential root causes of climate anxiety [87].

Higher scores on the CCAS significantly correlate with more psychological distress [94], general anxiety and depressiveness [94,96], pro-environmental intentions and policy support, less climate denial [96], climate concern, nature-relatedness, lower mindfulness, certain pro-environmental behaviors, information seeking and exposure [95], the New Ecological Paradigm, and lower self-efficacy [91]. Individual climate action was correlated with functional but not cognitive-emotional impairments, while climate activism was correlated with both subscales [94]. Additionally, women and younger age groups reported more climate anxiety [58,96]. Using a network approach, Heeren and colleagues proposed that the cognitive-emotional impairment subscale is central to the relationships among worry, climate change experiences, pro-environmental behaviors, and functional impairments [90]. Given this subscale, they also posit that cognitive-emotional impairment serves as a tipping point between pro-environmental behaviors or functional impairments resulting in avoidance of climate change action [90]. In Heeren and colleague’s directed acyclic graph model, worry and experience inform cognitive-emotional outcomes, which then inform a respondent’s action or lack thereof on climate change; however, they noted a paradoxical finding in worry and climate change experience in that these two factors were not found to have direct connections to functional impairments, suggesting a possible mediating effect somewhere in the model [90]. Predictors of increased climate change anxiety included younger age ranges [89,95], increased climate concern, generalized anxiety, nature-relatedness, and lower mindfulness [95]. Whitmarsh and colleagues report that those with an existing generalized anxiety disorder may be at higher risk of anxiety related specifically to a changing climate [95]. Mindfulness, inversely, was found to negatively correlate with reports of climate change anxiety, as they posit that it guards against invasive thoughts concerning climate change, fostering acceptance and, in some respondents, a greater propensity toward action [95]. People reported higher rates of worry about climate change and a lower prevalence rate for climate change anxiety [95]. The authors infer that this finding reflects high concern within their sample of United Kingdom citizens but also reflects little impact on the daily lives of their respondents [95].

### 1.7. Research Questions

Our literature review suggested that people possessed concerns about the consequences and impacts of climate change in a number of different but related domains. This may be especially the case given the recent extreme events of 2023 [5]. Beyond this, people may experience worry or anxiety about climate change. This literature naturally led us to wonder about the ways in which climate change impacts in different areas may intersect with the psychological responses people may experience. Thus, the authors conducted two studies to address the following research questions:

1. In a sample of university students in the southeastern United States, to what extent did people express: a. concern, b. worry, or c. anxiety as their predominant response to the potential impacts of climate change that included: (1) The ability to work and earn a livelihood in the future; (2) Effects upon future generations of people; (3) The occurrence of apocalyptic weather and climate events; (4) Effects on the physical environment; (5) Effects upon the living environment; and (6) Societal responses to climate change? Relatedly, to what extent did people indicate that they are unconcerned and have never thought about these six impacts or consequences of climate change?
2. To what extent were people’s responses to the six climate change impact areas related to measures of climate change worry, climate anxiety, and the perceived psychological distance from climate change? Further, to what extent is the willingness to engage in climate-sustainable behavior related to climate anxiety, worry, and psychological distance from climate change?

## 2. Study 1: Responses to Potential Climate Change Impacts

Our review of the literature suggested that a range of different climate change impacts and issues existed (e.g., ecosystem impacts and societal responses) [6,7,8,9,10,11,12,13,14] and that people may express different psychological responses (ranging from a lack of awareness to worry or anxiety) to these issues [58,68]. Thus, to speak simply of a person’s response to climate change overlooks the possibility that people may be aware of multiple and different climate change impacts and that such impacts may each evoke different psychological responses [61,62,63,64,65,66,67]. Because we could find no prior studies that empirically examined the possible range of climate change impacts and their associated responses, we undertook this exploration in our first study. We reasoned that such an exploration would provide a valuable orienting framework for quantifying the nature and frequency of concern, worry, and anxiety about climate change. Although a range of possible psychological responses exists, such as guilt, grief, and solastalgia, among others, e.g., [63,64], we focused here on worry and anxiety for three reasons. First, worry and anxiety are related to each other in that worry is a cognitive component of anxiety [76,77,78,79,80]. Second, published measures for worry and anxiety exist at this point, but such is not the case for other possible emotional responses specific to climate change [58,68]. Third, worry and anxiety function as primarily prospective and anticipatory psychological responses [76,77]. Although people may have worries or anxieties about past events, such past experiences are not necessary for worry or anxiety to arise, especially in the context of climate change, as Clayton noted [58,68].

### 2.1. Materials and Methods

#### 2.1.1. Participants and Procedures

The participant sample consisted of undergraduate and graduate students at a large state university located in the southeastern United States. Participation in the study was voluntary. The incentive for the study (for participants and non-participants) was entry into a randomized drawing to receive one of several Amazon gift cards of USD 50. The participants were sent an email that briefly described the study in a general way so that the potential for selection biases would be limited and that solicited their participation. The survey items were implemented via the Qualtrics platform. The study procedures and survey were reviewed and approved by the Institutional Review Board at the first author’s university (Approval: STUDY00005042, MOD00009609).

#### 2.1.2. Survey

The Qualtrics survey consisted of items to assess peoples’ responses to the six climate change impact areas and included five demographic information items, which were presented to the respondents at the end of the survey. For each of the six categories of climate impacts (1. effects upon livelihood, 2. future generations, 3. physical setting, 4. living systems, 5. effects of apocalyptic events, and 6. societal responses), the respondents were asked to indicate the one response option that best described their position about the possible impact of climate change at the time. There were four possible options for the category of impacts under consideration: 1 have not been thinking about the impact of climate change, 2. are concerned about the impact of climate change, 3. are worried about the impact of climate change, and 4. are anxious about the impact of climate change. The instruction set that accompanied the first item in each impact area read as follows:

*The next items ask you to indicate your position on different possible aspects of climate change. For the response choices, **concern** means that you think it is important and/or that you care about that aspect of climate change. **Worried** means that you have concerns about climate change that are troubling to you and that give rise to negative thoughts or feelings; worry is realistically based and usually is temporary. Finally, **anxious** means that you experience troubling negative thoughts and that these are often accompanied by bodily symptoms of being upset (e.g., butterflies in the stomach, nervous, twitchy, faster breathing or heart rate, sweating, etc.). Anxiety may last for a longer time, can generalize to other things, and can make it difficult to complete daily tasks such as work or school.*

These definitional anchors for worry and anxiety are consistent with the literature in affective science that sees worry as consisting primarily of beliefs or cognitions that are emotionally troubling but transient in time [73,74,75,76]. Anxiety responses encompass worry and additionally involve increased autonomic arousal and accompanying bodily symptoms (i.e., butterflies in the stomach), as discussed above in Section 1.6 [78,79,80].

If a respondent indicated that they had *not thought about* the impact of climate change or were just *concerned* about the impact, then the survey advanced to the items for the next impact area. If a respondent indicated that they experienced either worry or anxiety about a particular aspect of climate change, however, then an additional item appeared that asked them to use a fully-anchored five-point rating scale to indicate the magnitude of their worry or anxiety (1 = *Not at all worried/anxious* to 5 = *Extremely worried/anxious*). We programmed the survey to randomize the order in which the climate impact categories were presented to the respondents. Finally, the demographic items were presented after all the climate items and elicited information about the respondents’ age, gender, and ethnic identifications. We assessed political orientation with an eight-point rating scale (0 = *Extremely Liberal* to 7 = *Extremely Conservative*). The survey required approximately five minutes to complete.

#### 2.1.3. Data Analysis

We used the R Statistics Package for the data analyses in Study 1 [98]. Given the descriptive and exploratory nature of the study, we primarily calculated descriptive statistics and prepared graphs to depict the relationships for the possible range of responses to each of the six climate impact areas. We performed chi-square analyses to examine whether relationships existed among the participants’ categories of race, gender identification, and political orientation. Similarly, we performed chi-square analyses to assess the extent to which peoples’ psychological responses for the six impact areas were related to gender or race. Finally, we performed an analysis of variance to compare the mean levels of reported anxiety and worry responses for the six categories of climate change impacts.

### 2.2. Results

#### 2.2.1. Sample Characteristics

The participants were 506 undergraduate and graduate students. The sample contained 136 men and 356 women, 12 of whom indicated a nonbinary or gender-fluid identification, and two people who did not indicate their gender identification. The participants ranged in age from 18 to 60 years, *M* = 23.8 years, *Mdn* = 21 years, *SD* = 7.2 years. The racial composition of the sample was 62.7% White, 10.8% Asian, 4.8% Black, 4.6% Hispanic, 1.4% Indian subcontinent, and 13.8% Multiracial or another race. Eleven people did not provide information about their race (2%). In this regard, the sample generally reflected the demographics of the students’ university. Approximately 55.5% of the participants were liberal or liberal-leaning in their political affiliation. Overall, 25.5% of the sample were politically moderate, while 19.0% were conservative or conservative-leaning in their political affiliation. The distribution of participants according to race did not depend upon their gender identification. Finally, the mean scores on political orientation did not differ in a statistically significant way according to either the participants’ race or gender identification.

The participants’ university was in the state of Georgia in the southeastern United States. This region generally is susceptible to hurricanes, tropical storms, and flooding from these storms in the summer and early fall months. The region also regularly experiences severe thunderstorms, tornadoes, and flooding in the spring and summer [4]. From 2020 to 2023, the state of Georgia experienced impacts from 34 events (primarily hurricanes, tropical storms, and severe thunderstorms) that caused at least one billion dollars of damage each [4]. Thus, the participants were drawn from an area that can experience climate-related weather extremes.

#### 2.2.2. Psychological Responses to the Six Categories of Climate Change Impacts

**Figure 1:**
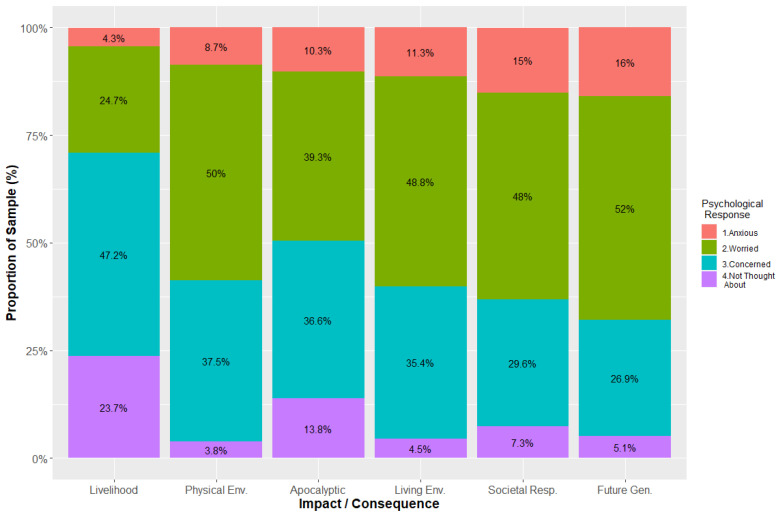
Psychological Responses to Six Climate Change Impacts and Consequences (n = 506).

Figure 1 shows the distribution of possible responses (Anxious, Worried, Concerned, or Not Thought About) for the six categories of climate change impacts. The categories of climate change concern are ordered in Figure 1, with the most worrisome or anxiety-related impacts appearing at the right. Overall, peoples’ psychological responses were related to the category of the climate change impact (or consequence), *Χ^2^* (N = 506, df = 15) = 296.9, *p* < 0.0001. Apart from the impacts of climate change on work and livelihood, the modal psychological response was one of worry (troubling, negative thoughts and feelings). The proportion of people who indicated worry as a modal response ranged from 24.7% (effects of climate change upon livelihood) to 52% (effects of climate change upon future generations of people). There were between 3 to 4 people who expressed worry about climate change impacts for every person who reported anxiety as their modal response.

The second most frequent response across the six categories of impacts was one of concern (i.e., an important and cared-about consequence of climate change). Anxiety (negative thoughts and bodily symptoms of being upset) about climate change impacts increased from 4.3% of the participants (for effects upon work and livelihood) to 16% regarding the consequences of climate change for future generations. Approximately one-quarter of the respondents had not thought about the impacts of climate change on work and livelihood. Similarly, 13.8% of them had not thought about the impacts of apocalyptic (severe/extreme weather) events as impacts of climate change. People reported the most distress (worry or anxiety) concerning societal responses (or the lack of them) in preparing for climate change impacts and about the consequences of climate change for future generations of people.

The participants’ psychological responses were not related to their gender (male and female) across the six climate change impact areas; because of their smaller number, we did not include people with gender-fluid/nonbinary in this analysis. We did observe, however, that for the impacts of climate change on the physical environment (*Χ^2^* (N = 492, df = 3) = 13.26, *p* < 0.004) and for the effects of climate change on future generations of people (*Χ^2^* (N = 492, df = 3) = 13.49, *p* < 0.004) that proportionately more men than women reported that they had either not thought about the respective climate impacts or that they were only concerned about the impact.

We observed that the participants’ psychological responses were related to their political orientation, *Χ^2^* (N = 506, df = 6) = 307.97, *p* < 0.0001. Approximately 68% of the respondents that were conservative or conservative-leaning (19.0% of the sample of 506) indicated that they either had not thought about the impacts or consequences of climate change or that they were only concerned about such impacts. In contrast, approximately 65% of the respondents who were liberal or liberal-leaning (55.5%) indicated that they were either worried or anxious about the impacts of climate change. People expressing a politically moderate orientation (25.5%) reported a more even distribution of psychological responses. Finally, we did not observe any noteworthy relationships between race with psychological responses for the six climate impact areas.

#### 2.2.3. Levels of Anxiety and Worry for the Categories of Climate Change Impacts

**Figure 2:**
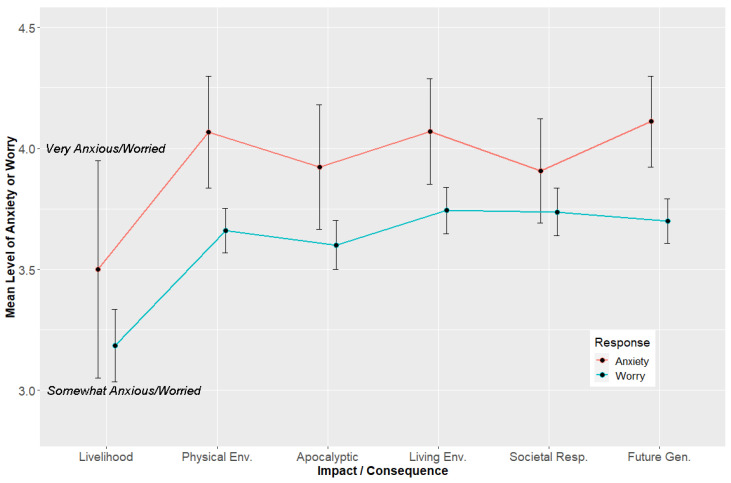
Mean Values of Worry and Anxiety (with 95% CI) for the Six Climate Change Impact Areas.

The participants who indicated that their modal response for a given climate change impact or consequence was either anxiety or worry were subsequently asked to rate their levels of these psychological responses. This naturally gives rise to the questions of (1) how the magnitudes of rated worry and anxiety compare with one another and (2) whether these responses differ across the categories of climate impacts and consequences. Figure 2 depicts the mean values of rated anxiety or worry for the six impact areas. The graph also includes 95% confidence intervals around each mean. The confidence intervals are somewhat wider for the anxiety means because of the smaller numbers of respondents who indicated that this was their primary response to each category of impact (see Figure 1).

We performed a two-way between-subjects analysis of variance to examine the patterns of differences among the means for anxiety and worry. Political orientation (using the 0–7 point rating scale) was also included as a covariate in this analysis. Although people provided repeated anxiety or worry ratings for the six impact areas, the majority of respondents provided a mixed profile of both anxiety and worry ratings when considering all six impact areas; for this reason, we treated the impacts and consequences of climate change as a between-subjects factor in the analysis of variance.

As depicted in Figure 2, there was a main effect for response (anxiety or worry), *F* (1,1596) = 31.52, *p* < 0.0001, *η^2^* = 0.0193, 90% CI: 0.009 to 0.032. When people indicated that their modal response was one of anxiety, they reported that they were very anxious and to a significantly greater degree (statistically) compared to the levels of worry that people expressed. Figure 2 shows that people were more anxious than they were worried about the impacts of climate change on the physical and living environments, the occurrence of apocalyptic weather events, and the impacts of a changed climate upon future generations of people. The mean levels of anxiety and worry ratings did not differ significantly concerning the impacts of climate change on the ability to work and earn a livelihood. People also expressed comparable levels of worry or anxiety about societal responses to climate change. The levels of worry that people exhibited qualitatively fell between being somewhat worried and very worried (see Figure 2).

We also observed a main effect in worry and anxiety responses according to the type of climate change impact, *F* (5, 1596) = 13.18, *p* < 0.0001, *η^2^* = 0.039, 90% CI: 0.023 to 0.053. The primary contributors to this statistically significant result were the somewhat lower ratings for anxiety (*M* = 3.5) and worry (*M* = 3.2) for the impact of climate change on the respondents’ abilities to work and earn a livelihood. These mean values were significantly lower than for the remaining levels of worry or anxiety across the other areas of impact or consequence. Other than this finding for livelihood impacts, the levels of anxiety or worry largely did not differ according to the category of impact.

The covariate of political orientation was a statistically significant contributor to the magnitudes of anxiety or worry that people expressed, *F* (1, 1596) = 94.86, *p* < 0.0001, *η^2^* = 0.053, 90% CI: 0.039 to 0.075. Across the six climate change impact areas, political orientation was negatively correlated with the magnitudes of reported anxiety or worry, *r* = −0.12, *p* < 0.0001. This means that increasing levels of conservative political orientation were associated with lower levels of anxiety or worry about an impact area.

### 2.3. Discussion of Study 1

The results from our first study were noteworthy in several respects, the first of which is that people in our sample exhibited different types of psychological responses depending upon the type of anticipated climate impact or consequence. The respondents expressed the most distress about societal responses to climate change and about the effects of climate change on future generations of people. Given the emphasis on this latter climate change impact area, it was somewhat unexpected that the participants expressed significantly less distress about climate change impacts on their abilities to work and pursue a livelihood. One possible explanation for this is that our sample of university students may have experienced the challenges of completing their education and entering the workforce as a more immediate concern, one that overshadowed the possible impacts of climate change.

A second noteworthy finding concerned the prevalence and magnitude of anxiety and distress in the Study 1 sample. Although a larger proportion of people reported worry about climate change within each of the six impact or consequence areas, those people who reported a response of anxiety experienced a significantly greater amount of anxiety—and distress from it—compared to those who reported a response of worry. This pattern was rather uniform when considering the six areas of climate change impacts. This result is consistent with the literature that views worry as a cognitive or thought-based component of anxiety and, with the component of somatic arousal, that anxiety may be experienced as more distressing and possibly debilitating [76,77].

Third, the participants’ political orientation, assessed on a continuum from liberal to conservative in outlook, was a significant contributor in shaping the nature and magnitude of reported psychological responses to the climate change impact areas, as researchers have observed previously [68,99,100,101]. Being conservative or conservative-leaning was associated with proportionately greater responses of either not having thought about a climate change impact or being concerned about it. Conversely, a liberal political orientation was more frequently associated with worry or anxiety about climate change. The role of political beliefs here is consistent with its role in Hornsey and colleagues’ meta-analytic review of contributors to beliefs about climate change [101]. Given the larger proportion of the sample that reported liberal sentiments, it is possible that this contributed to the modal response of worry in most of the climate change impact areas. This result is consistent with results by Gregerson and her colleagues, who reported in a large sample of European residents that political orientation moderated the relationship between beliefs that climate change was occurring and worry about climate change [99]. People with conservative political ideologies were less likely to worry about climate change [99]. McCright theorized that in the case of North America, political efforts to maintain the industrial capitalist enterprise have led both to political divisions and to a balkanized media that contributes to misinformation and muted societal responses to climate change [100]. Perhaps in this regard, political beliefs—especially those that are more conservative—help people to rationalize away beliefs about climate change that would otherwise lead to worry and perhaps subsequently to action about climate change. Beyond this, the contributions of political orientation to peoples’ responses to climate change point to the need for additional research in this area. Does political orientation, and relatedly, the politicization of climate change, contribute to people believing that the impacts do not exist or that the magnitude of the impact has been overstated? Similarly, does political orientation play a more direct role in affecting or minimizing the magnitude of concern, worry, and/or anxiety that people experience?

Finally, we did not observe differences in modal types of psychological responses to the six impact areas according to gender. This result was expected to some extent, even with the single items for worry and anxiety in the six areas, because gender differences did not emerge as significant in previous research on climate change worry [68] or in Clayton’s original work on climate anxiety [58].

## 3. Study 2: Relationships of Climate Worry, Anxiety, and Psychological Distance with Responses to the Six Climate Change Impact Areas

Although the results from Study 1 suggested that more people expressed concern, worry, or anxiety about the impacts of climate change on future generations and about societal responses to climate change, these categories each encompass somewhat longer, even distal, timeframes. In contrast, proportionately fewer participants in Study 1 expressed concern, worry, or anxiety about the impacts of climate change on their livelihoods and abilities to work and about the occurrence of severe or extreme (apocalyptic) weather events caused by climate change; these latter areas could, arguably, represent consequences that may be closer to participants with respect to time and space. Thus, the contributions of psychological distance (Section 1.3 above) as an antecedent condition to levels of climate worry and anxiety are relevant. The findings from Study 1 gave rise to the questions that we explored in Study 2: to what extent do worry and anxiety in the six climate change impact areas correlate with existing measures of climate change worry and climate anxiety, and the perceived psychological distance from climate change? Further, to what extent is the willingness to engage in climate-sustainable behavior related to climate anxiety, worry, and psychological distance from climate change?

The second question in this study is an important relationship to assess given prior research [49,50,58,63,71]. Here, climate change worry may serve as a catalyst or impetus for engaging in sustainable actions [21,102]. As an intensified or enduring emotional condition, climate anxiety could potentially hinder one’s motivations or capabilities to act sustainably. Also, it is possible that climate anxiety could contribute to a feeling of immobility [57,58,63]. Albrecht’s theory and research on psycho-terratic syndromes include the concept of eco-paralysis. As a feeling state, eco-paralysis could impede one’s ability to meaningfully address environmental challenges [63]. Similarly, Clayton has observed that in developing her measurement of climate change anxiety, the scores of climate anxiety did not exhibit any correlation with sustainable behaviors; this may suggest a certain level of paralysis resulting from anxiety [57,58]. Thus, it is of interest to assess these relationships empirically in the current study.

### 3.1. Materials and Methods

#### 3.1.1. Participants and Procedures

The participant sample consisted of 308 undergraduate students at a large state university located in the southeastern United States. Participation in the study was voluntary. The participants were part of a research pool within a college of education and received credit in one of their classes as an incentive for participation. To enroll for the study, the participants viewed the study title and a brief, general description of it. If they chose to participate, they gave their informed consent and then viewed the study measures online. The study measures were implemented via the Qualtrics platform, which allowed us to randomize the order of measure presentation. The demographics information portion was presented after all measures and just before the participants received the debriefing statement online. The demographic items were identical to those used in Study 1. The study procedures and measures were reviewed and approved by the Institutional Review Board at the first author’s university (Approval: PROJECT00005498).

#### 3.1.2. Survey Items and Measures

The participants in Study 2 responded to the items assessing their psychological responses to the six impact areas and consequences of climate change using the same procedure that we employed in Study 1 (see Section 2.1.1 above). That is, if the participant indicated that they experienced worry or anxiety about an impact area, they received an additional item that asked them to rate the magnitude of their worry or anxiety level. In addition to these items and the demographic items (which were identical to those used in Study 1), the participants also completed the Climate Change Anxiety Scale (CCAS) [58], The Climate Change Worry Scale (CCWS) [68], and a measure to assess psychological distance from climate change (PDCC) [32]. The measures are described below.

##### Climate Change Anxiety Scale (CCAS)

The Climate Change Anxiety Scale was designed to measure the level of anxiety about climate change that people self-report in responding to 13 items, such as, “I find myself crying because of climate change” and “I write down thoughts about climate change and analyze them” [58]. Originally, eight of the 13 items were intended to assess cognitive-emotional impairment, and five items measured functional impairment related to climate anxiety [58]. The respondents use a five-point, fully-anchored rating scale, 1 = Never to 5 = Almost Always. Thus, scores on the CCAS can range from 13 to 65, with higher scores indicating higher climate-related anxiety. The CCAS contains six additional items that assess the person’s engagement with sustainable behavioral practices that may help mitigate climate change. One item, for example, is: “I try to reduce my behaviors that contribute to climate change”. The CCAS behavioral engagement subscale utilizes the same rating scale; scores can range from 6 to 30, with higher scores indicating a greater inclination to behave in a sustainable manner. The CCAS has been a valuable new tool for assessing the range of negative, anxiety-related emotions people experience about climate change [87,88,89,90,91,92,93,94,95,96]. Although Clayton and Karaszia observed that a two-factor model (cognitive-emotional impairment and then functional impairment) provided an optimal fit, several researchers have offered different factor models for the 13 items, ranging from one factor to perhaps more than two. [91,103].

In the present sample, we treated the 13 CCAS items as a single scale, following the results of Innocenti et al. [91]. We also administered the six items assessing behavioral engagement. We created a total climate change anxiety score and a total score for behavioral engagement. Our participants responded consistently to the 13 CCAS items, α = 0.94, 95% CI: 0.92–0.95. The behavioral engagement items of the CCAS resulted in a somewhat lower, but still acceptable, level of internal consistency, α = 0.82, 95% CI: 0.77–0.85.

##### Climate Change Worry Scale (CCWS)

The Climate Change Worry Scale was intended to assess the degree of troubling, disturbing thoughts that people can experience about climate change [68]. The CCWS consists of 10 items that inventory aspects of a person’s climate change worry, such as: “Thoughts about climate change cause me to have worries about what the future may hold” and “I worry that outbreaks of severe weather may be the result of a changing climate”. The CCWS utilizes a 5-point fully-anchored rating scale, 1 = Never to 5 = Always. The CCWS scores can range from 10 to 50, with higher scores indicating higher levels of worry about climate change. In Stewart’s initial study, people were able to respond to the CCWS items in an internally consistent manner (α = 0.95), and the items were associated with a single latent factor of climate change worry [68]. In addition, people responded reliably to the CCWS items over a two-week test-retest interval (*r* = 0.91). [68]. Innocenti et al. [84] assessed the psychometric properties of an Italian version of the CCWS and observed a single factor and good test-retest reliability. In addition, CCWS scores were highly correlated with scores on a measure of pro-environmental behavior. For the present study, the CCWS items exhibited good internal consistency (α = 0.95, 95% CI: 0.94–0.95).

##### Psychological Distance from Climate Change (PDCC)

We assessed psychological distance from climate change using eighteen items that Wang and her collaborators developed for this purpose [32]. We refer to these items as the PDCC scale, although in the original study, these items were called the PD1 [32]. The PDCC represents an extension and modification of a ten-item measure that appeared eleven years ago to assess perceived distance from climate change [104]. The PDCC contains items for each of the four areas of psychological distance: temporal, spatial, social, and hypothetical distance [27]. Thus, of the eighteen PDCC items, there are four each for spatial and social distance from climate change. There are three items each for temporal and hypothetical distance. There are four items that combine spatial and social distance with temporal and hypothetical distance. An example of a spatial distance item is: “I feel geographically far from the effects of climate change”. The temporal-spatial hybrid item is: “Climate change will not change my life or my family’s lives anytime soon”. Participants responded to the scale using a five-point fully-anchored rating scale: 1 = Strongly Disagree to 5 = Strongly Agree; some items are reverse-scored. In two studies, the PDCC items loaded primarily on a single factor, which suggested a single latent variable of psychological distance from climate change [32]. In at least one study, less psychological distance was associated with greater pro-environmental behavior [32]. We used the 18 items of the PDCC and summed the items (after reverse scoring the appropriate items) to produce a total score; the PDCC scores had a possible range of 18 to 90. Greater scores were associated with greater perceived distance from climate change impacts. With the present sample, the PDCC items exhibited an acceptable level of internal consistency, α = 0.91, 95% CI: 0.89–0.93.

#### 3.1.3. Data Analysis

We again used the R Statistics Package for the data analyses in this study [98]. We used R to calculate descriptive statistics, correlations, and linear regression. We used the *ltm package* to calculate the coefficient alpha (α) values and confidence intervals [105]. We used the *cocor package* to check for significant differences in correlations from a single group that shares a common variable [106]; within this package, we interpreted the *z* statistic from Meng, Rosental, and Rubin [107]. Finally, the *ppcor package* was used to calculate partial correlations [108].

### 3.2. Results

#### 3.2.1. Sample Characteristics

The participants were 308 undergraduate students that included 53 men, 247 women, and 8 who were nonbinary or gender-fluid in their gender identification. The participants ranged in age from 18 to 41 years, *M* = 21.0 years, *Mdn* = 21 years, *SD =* 2.0 years. The racial composition of the sample was: 73.4% White, 7.5% Black, 5.8% Hispanic, 5.2% Asian, and 8.4% Multiracial or another race. Approximately 47.2% of the participants indicated that they were liberal or liberal-leaning regarding their political orientation. Politically moderate participants made up 29.8% of the participants, and 23.0% indicated that they were conservative or conservative-leaning. Three participants did not indicate their political orientations. Identical to Study 1, the participants’ university was in the state of Georgia. This region generally is susceptible to hurricanes, tropical storms, and to flooding from these storms in the summer and early fall months. The region also regularly experiences severe thunderstorms, tornadoes, and flooding in the spring and summer [4]. Thus, the participants were drawn from an area that regularly experienced climate-related weather extremes.

#### 3.2.2. Relationships of Climate Change Worry and Anxiety with Psychological Distance from Climate Change

The intercorrelations of climate change worry, climate anxiety, psychological distance, and political orientation appear in Table 1. This table also provides the means and variances for the variables.

The correlations in Table 1 indicated that as the psychological distance from climate change increases, there were associated decreases in both climate anxiety and climate change worry; the performance of climate sustainable behaviors also decreases. The performance of climate-sustainable behaviors was positively associated with both climate anxiety and climate change worry (see Table 1). The correlation of sustainable behaviors with climate change worry was significantly larger than it was with climate anxiety, *z* = 4.42, *p* < 0.0001.

As political beliefs increased in the conservative direction, this was associated with increasing psychological distance from climate change. To investigate this further, we calculated partial correlations among the climate variables in Table 1, controlling for political orientation. The correlation values changed only slightly in magnitude and remained highly statistically significant. The correlations of psychological distance of climate change with climate anxiety and worry, respectively, were: *r_p_* = −0.20, *p* = 0.0004 and *r_p_* = −0.53, *p* < 0.0001. The correlation between climate anxiety and climate change worry decreased slightly, *r_p_* = −0.42, *p* < 0.0001. Thus, although political orientation was related to the climate variables in Table 1, it did not appear to affect the magnitude of relationships of psychological distance with the other climate variables substantially.

One of the research questions for this study was: to what extent is the willingness to engage in climate-sustainable behavior related to climate anxiety, worry, and psychological distance from climate change? We used scores on the CAS, the CCWS, PDCC, and political orientation in a linear regression model to predict the CAS behavioral engagement scores. Both climate change worry and psychological distance predicted climate sustainable behaviors, *F* (2, 305) = 43.34, *p* < 0.0001, *R^2^_adj_* = 0.212. Climate anxiety and political orientation were not statistically significant contributors to the regression. Climate change worry was positively associated with climate-sustainable behaviors, and psychological distance was negatively associated with such behaviors. The regression equation with standardized regression coefficients is given in Equation (1) below.

CCAS Sustainable Behaviors = 0.283∙(CCWS) − 0.245∙(PDCC)(1)

#### 3.2.3. Relationships of Climate Change Worry, Anxiety, and Psychological Distance with Responses to the Six Climate Change Impact Areas

The participants in Study 2 also indicated their modal response (anxiety, worry, concern, or not being aware or not having thought about) for each of the six climate change impact areas as described in Study 1. When indicating anxiety or worry, the participants were asked to supply a rating of the magnitude of the response (anxiety or worry) for the climate change area. Like the results from Study 1, there was a trend for people to report a response of worry more frequently than one of anxiety (see Table 2). Also consistent with results from Study 1, people more frequently reported worry or anxiety about the impacts of climate change on future generations and societal responses to climate change; they less frequently reported worry or anxiety about the impact of climate change on their livelihoods. Thus, the results generally supported those reported in Study 1.

Table 2 also shows the correlations of rated anxiety or worry magnitudes with climate anxiety, climate sustainable behaviors, climate change worry, and the psychological distance from climate change within each of the impact or consequence areas. Regarding anxiety, there was a trend for the correlations between rated anxiety in the impact areas to correlate more highly in magnitude with scores on the CAS than with climate change worry; this was especially the case for anxiety about impacts on the living environment (see Table 2). Some of the correlations involving anxiety ratings were not statistically significant because of the small number of people who indicated anxiety as their modal response for an impact area. The worry ratings that respondents provided for each of the six impact areas were significantly correlated with scores on the CCWS.

Psychological distance from climate change scores exhibited statistically significant correlations with ratings of worry magnitude in five of the six impact areas. The correlations ranged from −0.27 (not significant) for livelihood and ability to work to −0.43 for worry about the occurrence of climate-related apocalyptic weather events. The negative sign on the correlations conveyed that as psychological distance from climate change decreased, ratings of worry increased.

Finally, ratings of worry about climate change impacts on the living environment, upon future generations of people, and societal responses to climate change were each slightly but significantly correlated with the tendency to perform climate-sustainable behaviors (see Table 2). We calculated the partial correlations, removing the effects of psychological distance. Only one of the correlations of rated worry with sustainable behaviors remained statistically significant: societal responses to climate change, *r_p_* = 0.21, *p* = 0.01. Thus, within some of the climate change impact areas, consistent with what we reported in Equation (1), the psychological distance from climate change can contribute to engagement with climate-sustainable behaviors.

### 3.3. Discussion of Study 2

Study 2 was an exploratory inquiry to assess the extent of relationships among climate anxiety, climate sustainable behaviors, psychological distance from climate change, and political orientation. The results of this study were noteworthy because, to the authors’ knowledge, this represents the first investigation of climate anxiety and worry together with a measure of psychological distance from climate change. Both climate change anxiety and climate change worry were significantly associated with psychological distance from climate change. Greater perceived distance from climate change was associated with lower climate change anxiety and worry. The greater degree of association between psychological distance and climate change worry may have stemmed from the fact that both the PDCC and the CCWS assess thoughts and beliefs about climate change, whereas the assessment of anxiety in the CCAS may encompass arousal and bodily states associated with anxiety, which were not represented in the items of either the CCWS or PDCC.

The regression result (see Equation (1)) also was noteworthy for two reasons. Consistent with existing research, climate change anxiety was not associated with engagement with climate-sustainable behaviors as much as worry [49,50,58,71]. Perhaps the worry that accompanies the thoughts and beliefs about climate change, as contained in the CCWS items, provides motivation or impetus for engaging in sustainable behaviors [21,102]. As a more intense or enduring emotional state, climate anxiety may diminish one’s motivations or abilities to behave sustainably or perhaps contribute to a sense of paralysis, as described by Albrecht [57,58,63]. Similarly, Clayton has noted that in defining and developing her measure of climate change anxiety, climate anxiety scores were not correlated with sustainable behaviors; perhaps this implies some degree of paralysis due to anxiety [57,58]. An implication of this result is that messages to encourage sustainable behavior should be crafted such that they are motivating rather than overwhelming, especially as the latter could involve anxiety or fear.

Another interesting result from Study 2 was that the inclusion of psychological distance as a predictor of engaging in sustainable behaviors appeared to suppress the contributions of political orientation. To our knowledge, our study is the first to use the PDCC measure in relation to respondent political orientation. This is noteworthy because, in previous studies, political beliefs and orientations have been strong predictors of beliefs in climate change [99,100,101], its anthropogenic causes [99], and worry about climate change [68,99]. It is possible that the PDCC is reflecting the degree of engagement with climate change as a pressing societal issue as this is a component of different (liberal or conservative) beliefs. More research on the PDCC measure and political orientation appears warranted.

The correlational results conveyed in Table 1 were generally consistent with the pattern of results observed within the six climate change impact areas in Table 2. Namely, expressed worry about the impact area was correlated significantly and at a higher magnitude with psychological distance than was anxiety about the impact area. Similarly, for three of the six areas, expressed worry about the area was associated with engagement with sustainable behaviors.

The results from Table 2 also provided some discriminant validational evidence for the CCWS and, to a more limited extent, the CCAS. That is, the CCWS scores were significantly correlated with the ratings of worry within each of the six impact areas and were higher in magnitude than CCWS correlations with rated anxiety for each impact area. Similarly, the CCAS correlations with rated anxiety in each climate change impact area were higher in magnitude than was the rated level of worry, although the smaller number of people indicating anxiety as a modal response compared to worry resulted in the correlations not achieving statistical significance in most of the impact areas.

Overall, the results from Study 2 supported those in Study 1 that a larger proportion of the respondents indicated worry compared to anxiety as a psychological response to impacts or consequences in different areas. In both studies, worry and anxiety about the consequences of climate change for future generations was identified as the most pressing concern. Because the participant sample in Study 2 was smaller (N = 308) than in Study 1 (N = 506) and because the second sample came from a college of education research pool (compared to university-wide in Study 1), some differences in the responses to impact areas were noted. For example, Study 1 participants ranked concern about the physical environment lower than Study 2 participants, where 40% (of 308 participants) indicated worry about physical environment impacts.

## 4. General Discussion

In this article, we explored the ways that concern, worry, and anxiety [45,48,58,68] were related to various possible climate change impacts and consequences [6,7,8,9,10,11,12,13,14]. We also examined the associations of the psychological distance to climate change to climate change anxiety and worry, both generally and within the six impact areas that were the focus of our research. The results were noteworthy in several respects, the first of which was that peoples’ responses (not aware or thought about, concerned, worried, or anxious) differed according to the climate change impact areas. In both studies, we observed that the respondents more frequently expressed worry or anxiety about the impact of climate change on future generations [6]. Conversely, they less frequently expressed worry or anxiety about the impacts on their livelihoods and abilities to work [7,8]. This result was somewhat puzzling because one could reason that for university students, climate change impacts may be psychologically closer and more immediate on their abilities to work compared to the impacts upon future generations. Given the relatively young ages of most of the respondents in each study, it is possible that they viewed themselves, in part, as a generation that already has been affected by global climate change and disruption. This is a plausible explanation for the finding, given that the first IPCC Assessment Report appeared approximately 33 years ago [109]. In addition, these generations have been exposed to increasing media coverage of climate change and its impacts, which could affect their psychological distance from climate change [16,17,18,19]. Similarly, it is possible that finishing university studies and securing employment were experienced as greater and more immediate challenges for the participants than the contributions of climate change to their livelihoods [110].

These results may be unique to the location and demography of the participants in our studies, which were conducted in the southeastern United States. It would be informative to use the approach in this project (i.e., study the range of psychological responses to different categories of impacts that differ in the psychological distances) with samples that are diverse with respect to their geographic and climatic zones, economic, sociocultural, and political statuses, and their existing responses to climate change. Studies with such a diverse and/or international scope would be possible given that the Climate Change Worry Scale and the Climate Change Anxiety Scale have been translated into several different languages [58,68,84,85,87,91,92,93].

Second, we observed that across the different climate change impact areas, the participants more frequently reported worry compared to anxiety. This result could have occurred because worry is a component of different negative emotional experiences like generalized anxiety, dysthymia, and depression [76]. As a feature of several syndromes, it is understandable that worry may be more prevalent. This result is also consistent with prior clinical research that has reported more frequent experiences of worry than anxiety [67,68,69,70,71,72,73,74,75,76,77,78,79,80].

The relationships between worry and psychological distance with the CCAS behavioral engagement subscale accords with prior research that has documented that anxiety about climate change or the environment has not been related to pro-environmental behaviors [32,49,50,58]. Although some motive for such behaviors is necessary, anxiety may not provide the impetus for climate-sustainable behaviors and may be associated with paralysis or hopelessness about engaging in pro-environmental behaviors [57,58,63,95]. Engaging in collective action with other people may help to ameliorate some of the negative effects of climate change anxiety and build a sense of agency [94].

Third, our project was the first to use the PDCC measure developed by Wang et al. to assess the relationships of psychological distance from climate change with published measures of climate change worry and anxiety [32,58,68]. The pattern of results—that increasing psychological distance from climate change was associated with decreasing worry and anxiety—provides convergent validity support for the PDCC. Further research to establish the psychometric properties of the PDCC items and to examine their functionality as a self-report measure appears warranted.

Relatedly, it was interesting to observe that although the PDCC total scores were only modestly correlated with political affiliation (see Table 1), psychological distance was a stronger predictor of sustainable behavior engagement on the CCAS than political orientation. In fact, political orientation became statistically non-significant in the regression we conducted (see Equation (1)). This result, along with the result that when controlling for political orientation, the partial correlations of variables in Study 1 were largely unchanged, suggested to us that psychological distance may be a more precise indicator of the effects of political orientation upon climate change beliefs, feelings, and behaviors. That is, at least for our sample of students within the southeastern United States, it may not be so much the effects of the totality of one’s political beliefs but the effects such beliefs have upon the psychological proximity of climate change. This is an important finding because psychological distance from climate change can be easily assessed and could, perhaps, function as a proxy for the effects of political beliefs with samples and settings where it may not be possible or advisable to inquire about political orientation [111,112].

### Limitations

Our research is limited in several ways, the first of which concerns the reliance upon samples of students from a large state university in the southeastern United States. Although this university serves a wide demographic of domestic and international students and possesses students across the range of socioeconomic statuses, the research reported here is not representative of older individuals who live in different geographic and climatic zones who may be vulnerable to different or more severe impacts from climate change. Although the method we used may be promising in work with more diverse samples, the results we observed are likely affected by the nature of the samples we relied upon, and this necessarily limits the generalizability of the results. The sample, however, was drawn from a region that regularly experiences severe and extreme weather events that can impact life and affect people psychologically [4,38].

A second limitation is that the measures we used depended upon the participants’ self-reports of their experiences of concern, worry, and anxiety. Although we provided definitions of worry and anxiety, it is possible that some people may not be able to reliably determine what kinds of psychological responses they experience to different prospective climate change impacts. This unreliability in self-report undoubtedly introduces errors in the results and can limit their utility. Similarly, although we inquired about political orientation, we did not have any way to verify the accuracy of what the participants self-reported for this variable.

The third limitation of our work is that we did not fully assess the possible spectrum of psychological responses to climate change. Our focus was on responses that are primarily anticipatory and prospective in nature: concern, worry, anxiety. That is, as people anticipate different climate change impacts in the near or distant future, how do they respond? Other psychological responses are possible, especially for people who already have experienced the impacts of a changed climate system. Some of these responses include grief and mourning [51,64,65], solastalgia (longing for the return of a healthy planet) [63], frustration and anger [61], guilt, fear, and trauma [51,62]. In addition, we asked the participants to indicate a modal, single response for each of the impact areas. To some extent, this instruction was justified because concern, worry, and anxiety are related responses [76,77,78,79,80]. It is possible that people could experience a combination of emotions such as anxiety, grief, and anger, among others. It would be especially informative to assess these multiple responses in samples of people who already have experienced impacts from climate change. Nonetheless, our study was useful in revealing the prevalence of concern, worry, and anxiety for different possible climate change impact areas, as these have been delineated in the literature [1,6,7,8,9,10,11,12,13,14].

## 5. Conclusions

The continuing occurrence of severe and extreme weather events that have been attributed to a changed climate system and the widespread dissemination of the impacts of these events in the media can lead people to experience concern, worry, and anxiety (among other responses). Our explorations revealed that people more frequently expressed worry than anxiety across the impact areas of climate change. Except for one area (impacts on the ability to work and livelihood), the modal response was one of worry. People were more frequently worried and anxious about the effects of climate change on future generations and about societal responses (or lack of a response) to climate change and expressed such responses much less frequently for impacts of climate change on their abilities to work. In addition, the levels of worry that people expressed (Study 1) were significantly higher than the anxiety people reported when anxiety was their modal response. In Study 2, we observed that both climate change worry and anxiety were negatively correlated with psychological distance from climate change. Overall, climate change worry and psychological distance significantly predicted climate-sustainable behaviors. Our study was among the first to use developed measures of climate change worry, anxiety, and psychological distance to examine peoples’ responses across some of the possible impact and consequence areas of climate change.

## Figures and Tables

**Table 1:** Pearson Correlations of Psychological Distance, Climate Anxiety, Climate Change Worry, and Political Orientation (N = 308).

Scale	1	2	3	4	5
1. Psychological Distance from Climate Change (PDCC)	--				
2. Climate Change Anxiety Scale (CCAS)	−0.22	--			
3. CCAS—Behavioral Engagement	−0.40	0.17	--		
4. Climate Change Worry Scale (CCWS)	−0.55	0.45	0.42	--	
5. Political Orientation	0.30	−0.14 *	−0.20	−0.32	--
Mean	44.2	24.6	21.8	22.7	2.9
Variance	118.0	45.7	21.2	87.2	8.4

Note: N = 305 for political orientation because of missing values for this variable. * The significance level, *p*, was 0.012. For all other correlations, *p* < 0.0001.

**Table 2:** Pearson Correlations of Climate Anxiety, Sustainable Behaviors, Worry, and Psychological Distance with Rated Anxiety and Worry in the Six Climate Change Impact Areas (N = 308).

Psychological Response to Climate Change Impact or Consequence Area (Percentage of 308 Respondents)		Climate Anxiety Scale	Climate Sustainable Behaviors	Climate Change Worry	Psych. Distance from Climate Change
**Livelihood and Work** **Ability**	(15.6%) Worry	0.06	0.00	0.36 *	−0.29
	(3.9%) Anxiety	--	--	--	--
**Impacts on Physical** **Environment**	(40.9%) Worry	0.11	0.15	0.40^†^	−0.37 ^†^
	(6.8%) Anxiety	0.36	−0.28	0.30	−0.15
**Occurrence of Apocalyptic Events**	(31.8%) Worry	0.12	0.09	0.47^†^	−0.46 ^†^
	(6.5%) Anxiety	0.49	0.00	0.24	−0.06
**Impacts on Living** **Environment**	(11.4%) Worry	0.17 *	0.25 *	0.51 ^†^	−0.39 ^†^
	(8.8%) Anxiety	0.48 *	0.12	0.31	−0.05
**Societal Responses to** **Climate Change**	(38.6%) Worry	0.11	0.26 *	0.47 ^†^	−0.38 ^†^
	(8.8%) Anxiety	0.23	0.05	0.18	−0.18
**Impacts/Effects on** **Future Generations**	(47.7%) Worry	0.19 *	0.21 *	0.49 ^†^	−0.42 ^†^
	(9.7%) Anxiety	0.12	0.04	0.34	−0.19

Note: -- indicates that we did not believe that (n = 12, 3.9%) would yield meaningful correlation estimates and therefore omitted them from the table. * indicates a statistically significant correlation, *p* < 0.05. ^†^ indicates a statistically significant correlation, *p* < 0.0001.

## Data Availability

For data availability, please contact the first author.
